# Nitroxide—HMP—Protects Human Trophoblast HTR-8/SVneo Cells from H_2_O_2_-Induced Oxidative Stress by Reducing the HIF1A Signaling Pathway

**DOI:** 10.3390/antiox12081578

**Published:** 2023-08-08

**Authors:** Diana Pintye, Réka Eszter Sziva, Maxim Mastyugin, Marianna Török, Sonako Jacas, Agnes Lo, Saira Salahuddin, Zsuzsanna K. Zsengellér

**Affiliations:** 1 Department of Medicine, Beth Israel Lahey Health, Beth Israel Deaconess Medical Center, Harvard Medical School, Boston, MA 02115, USA; dpintye@bidmc.harvard.edu (D.P.); dsziva@bidmc.harvard.edusjacas@bidmc.harvard.edu (S.J.); alo1@bidmc.harvard.edu (A.L.); 2 Department of Obstetrics and Gynecology, Semmelweis University, 1085 Budapest, Hungary; 3 Department of Chemistry, University of Massachusetts, Boston, MA 02125, USA; maxim.mastyugin001@umb.edu (M.M.); marianna.torok@umb.edu (M.T.); 4 Department of Obstetrics and Gynecology, Beth Israel Lahey Health, Beth Israel Deaconess Medical Center, Harvard Medical School, Boston, MA 02115, USA; ssalahud@bidmc.harvard.edu

**Keywords:** preeclampsia, oxidative stress, mitochondria, sFLT1, nitroxides, hypoxia, trophoblast cells

## Abstract

Preeclampsia (PE) is a pregnancy-specific syndrome affecting 5–7% of patients. There is no effective treatment available. Early abnormal placental development is associated with oxidative stress (OS) and a release of reactive oxygen species (ROS) in the placenta. This phenomenon leads to downstream signaling, Hypoxia Inducible Factor 1A (HIF1A) stabilization and transcription of the anti-angiogenic factors soluble fms-like tyrosine kinase 1 (sFLT1) and soluble endoglin (sEng), which are known to cause endothelial and trophoblast dysfunction and cardinal features of PE: hypertension, proteinuria and, in severe cases, eclampsia. We tested whether 3-(Hydroxymethyl)-1-oxy-2,2,5,5-tetramethylpyrrolidine (HMP)—a nitroxide-type antioxidant molecule—can reduce placental OS and mitigate PE symptoms in vitro. We induced OS in human trophoblast (HTR-8/SVneo) cells with hydrogen peroxide (H_2_O_2_) and assessed whether modulating cell redox function with HMP reduces cell injury, mitochondrial stress and HIF1A and sFLT1 production. Pre-treatment with HMP reduced mitochondrial-derived ROS production, restored LC3B expression and reduced HIF1A and sFLT1 expression in H_2_O_2_-exposed HTR-8/SVneo trophoblast cells. HMP improved the mitochondrial electron chain enzyme activity, indicating that a reduction in OS alleviates mitochondrial stress and also reduces anti-angiogenic responses. In reducing placental trophoblast OS, HMP presents a potential novel therapeutic approach for the treatment of PE. Future investigation is warranted regarding the in vivo use of HMP.

## 1. Introduction

Preeclampsia (PE) is one of the most serious complications of pregnancy, characterized by hypertension and proteinuria occurring after 20 weeks [1]. It is associated with significant maternal, fetal and neonatal morbidity and mortality [2]. Classically, PE is defined by de novo maternal hypertension (>140/90 mmHg systolic/diastolic blood pressure) and proteinuria (>300 mg/24 h). In severe cases, the mother may develop comorbidities such as hepatic alterations (HELLP syndrome), edema, disseminated vascular coagulation (DIC) and eclampsia, which specifically affects the brain. For the fetus, the main complications associated with PE include growth restriction leading to low birth weight (1/3 of cases), prematurity and fetal death [3,4,5,6]. 

Currently, other than a preventive intake of low-dose aspirin (≥100 mg) in high-risk women before the 16th week of their pregnancies [7], lowering high blood pressure with anti-hypertensive agents and premature termination of the pregnancy (C-section or parturition induction) in developed PE, there are no targeted medical treatments for this disease yet. 

While the etiology and pathogenesis of PE is elusive, it is currently believed that placental ischemia, due to impaired spiral artery remodeling, is the primary culprit. It has been proposed that the ischemic state will cause oxidative stress (OS) and impair endothelial and trophoblast function, which may contribute to the pathogenesis of PE. Significant reactive oxygen species (ROS) production in the placenta in PE pregnancy has also been reported [8,9,10,11,12,13,14,15,16,17,18,19]. Furthermore, the mitochondrial electron transport chain enzyme cytochrome C oxidase (COX) is diminished in the syncytiotrophoblast cells of the placenta, implicating mitochondrial damage/dysfunction as a potential contributor to the pathogenesis of PE [9]. The release of ROS and/or reactive nitrogen species (RNS) can stabilize Hypoxia Inducible Factor 1A (HIF1A), which will induce transcription of anti-angiogenic factors such as soluble fms-like tyrosine kinase 1 (sFLT1) and soluble endoglin (sEng) [20,21,22,23,24,25,26,27]. The anti-angiogenic factors are released into the maternal circulation, and their actions are thought to disrupt the maternal endothelium and result in hypertension, proteinuria and other systemic manifestations of PE [28,29,30,31].

Given that placental oxidative stress may be an early trigger in the pathogenesis of preeclampsia, therapies that counteract the antioxidant pathways have been proposed as treatments. The first attempts in clinical trials have shown that vitamin C and vitamin E therapy have only modest benefits on PE symptoms [32]. Therefore, there is interest in developing alternate strategies to reverse oxidative stress in the placenta. Targeted antioxidants have been used in models of ischemia–reperfusion injury [33,34,35,36] and are attractive prospective treatments as preeclamptic tissue is characterized by profound mitochondrial oxidative stress [37,38].

3-(Hydroxymethyl)-1-oxy-2,2,5,5-tetramethylpyrrolidine (HMP) is a potent redox catalyst, by virtue of the pyrrolidine nitroxide functional group, which acts as a degradation catalyst of ROS [39,40,41]. The spectrum of ROS degradation by HMP includes (i) superoxide ion/O_2_^−^ dismutation, (ii) catalase-like activity (detoxifying H_2_O_2_) and (iii) peroxy-nitrite ion/ONOO^−^ decomposition. HMP will target the imbalance in both oxygen-centered and nitrogen-centered free radicals, thereby acting on a major component underlying the pathophysiology of preeclampsia. There are no current therapies available that address these contributors to the early development of PE. Furthermore, there are no proposed/investigational therapies that address mitochondrial redox balance. 

We hypothesized that HMP will exhibit effective antioxidant properties as well as proper membrane permeability, which will result in promising lead compounds that can serve in practical drug development. Here, we demonstrate that HMP exerts mitochondrial-protective effects, reduced oxidative stress, reduced HIF1A production and subsequent prevention of the anti-angiogenic response of H_2_O_2_-exposed HTR-8/SVneo trophoblast cells. These effects were compared with other well-known antioxidants–N-acetyl-cysteine (NAC) and MitoTEMPO (MT) [42,43,44].

## 2. Materials and Methods

### 2.1. Materials

HMP: 3-(Hydroxymethyl)-1-oxy-2,2,5,5-tetramethylpyrrolidine was purchased from Toronto Research Chemicals (Toronto, ON, Canada). MitoTracker™ Green FM and MitoSOX™ Red were purchased from ThermoFisher Scientific (Waltham, MA, USA). Phosphate-buffered saline (PBS), RPMI medium, fetal bovine serum (FBS), trypsin and penicillin-streptomycin were purchased from Gibco, Invitrogen (Carlsbad, CA, USA).

### 2.2. Antioxidant Assay

The Oxygen Radical Antioxidant Capacity (ORAC) radical scavenging assay was carried out as described earlier after minor modifications [45,46]. For the ORAC preparation, 200 mL of 75 mM phosphate buffer at pH 7.4 was incubated for 2 h at 37 °C. A 0.08 μM fluorescein solution was prepared in this phosphate buffer. Stock solutions of the control compounds (ascorbic acid, Trolox, and MitoTEMPO) were prepared in DMSO to a concentration of 10 or 50 mM. The experimental compound (HMP) was also prepared to a concentration of 10 or 50 mM in DMSO. The compound stock solutions were further diluted in DMSO through serial dilution to reach the needed concentrations. All the compound stock solutions were diluted with ethanol by a dilution factor of 125. Then, 25 μL of each compound was added to a black flat-bottom 96-well plate in triplicate. The 2,2′-azobis(2-amidinopropane) dihydrochloride (AAPH) radical solution was prepared by dissolving AAPH in cold (4 °C) phosphate buffer to a concentration of 153 mM. Control sets with AAPH and fluorescein with the appropriate amount of DMSO in ethanol were used to determine background absorbance. Sets with no AAPH (cold phosphate buffer added instead) and only fluorescein were used as a positive control. Sets with AAPH and no fluorescein (phosphate buffer added instead) were used as a negative control. Then, 150 μL of the fluorescein solution and 25 μL of the AAPH solution were added to each well of the 96-well plate, except for the negative and positive control wells, respectively. The fluorescein solution was added first, and the plate was incubated for 15 min at 37 °C with the AAPH being added after the incubation. A SpectraMax i3x UV-Vis/fluorescence plate reader was set to 37 °C with the excitation and emission wavelengths at 485 nm and 520 nm, respectively. The plates were read with the SoftMax Pro 5 software (Molecular Devices) to assess the scavenging of the AAPH radical by the investigated compounds. Readings were collected every 2 min for 60 min. The data were processed using the equations below using the following parameters:

$$Net\;AUC=0.5+\sum_{0-29}\frac{f_{i}}{f_{0}}+\left(0.5\times \frac{f_{30}}{f_{0}}\right)$$

f_i_ is the fluorescence intensity at reading 0–29. f_0_ is the fluorescence at reading zero. f_30_ is the fluorescence at reading 30.

$$Percent\;Radical\;Scavenging=\frac{\left({Net\;AUC}_{t}-{Net\;AUC}_{c}\right)}{{(Net\;AUC}_{f\_max}-{Net\;AUC}_{c})}\times 100$$

The Net AUC_c_ is the net area under the curve for the control sample with no compound. The Net AUC_t_ is the area under the curve for the test sample while the Net AUC_f_max_ is the area under the curve for the maximum fluorescence sample where no AAPH was added (positive control). 

### 2.3. Cell Culture Studies

Human trophoblast HTR-8/SVneo cells were obtained from ATCC (American Type Culture Collection, Manassas, VA, USA) and were cultured in RPMI medium supplemented with 5% fetal bovine serum and 1% penicillin-streptomycin in a humidified incubator containing 5% CO_2_ at 37 °C. Cells in the H_2_O_2_-treated (“Control”) group were treated with 100 μM H_2_O_2_ alone. Cells in the HMP + H_2_O_2_-treated (“Treated”) groups were pre-treated with 1–200 μM HMP for 30 min, respectively, and then they were treated with 100 μM H_2_O_2_ for 24 h. Cell culture supernatants were collected at the end of the experiment and stored at −20 °C until assayed.

### 2.4. Cell Viability Assay

Following treatment with antioxidant and H_2_O_2,_ cell viability was determined with a Cell Counting kit-8 (CCK-8) assay (Dojindo Molecular Technologies, Inc., Shanghai, China) and read on a Bio-Rad microplate reader at 450 nm, as previously described [8].

### 2.5. Biochemical Measurements

HTR-8/SVneo cells were seeded in 48-well plates (Nalgen Nunc International, Rochester, NY, USA) and incubated at 37 °C in a 10% CO_2_ humidified incubator at 37 °C overnight. The next day, cells were subjected to H_2_O_2_ treatment for 24 h, along with various concentrations of HMP. After 24 h, the cells were incubated with MitoTracker™ Green FM (#M7514 ThermoFisher Scientific) and MitoSOX™ Red (#M36008 ThermoFisher Scientific) fluorogenic dyes and the specific fluorescence of the various dyes was visualized and photographed using an inverted EVOS^®^ FL Imaging System (Advanced Microscopy Group, Mill Creek, WA, USA) [8].

### 2.6. HIF1A Immunofluorescence

Immunofluorescence was used to determine the nuclear translocation of HIF1A. Briefly, HTR-8/SVneo cells were grown on Lab-Tek slides and were fixed in 4% paraformaldehyde, permeabilized with 0.5% Triton X-100, blocked with 1% BSA and then incubated with anti-HIF1A antibody (Alexa Fluor^®^ 488 Anti-HIF-1 alpha antibody [EP1215Y] ABCAM#ab190197 1:100 dilution) overnight at 4 °C. The nuclei were stained with DAPI and then the slides were viewed under an inverted EVOS^®^ FL Imaging System (Advanced Microscopy Group). 

Morphometric measurements were generated from fluorescence microscopy images for MitoSOX™ Red, MitoTracker™ Green FM and HIF1A and light microscopy images for trophoblasts COX enzyme chemistry with an original magnification of 20×. Morphometric measurements were performed using ImageJ software version 1.53t (National Institute of Health [NIH], Bethesda, MD, USA; http://imagej.nih.gov/ij). Staining intensity was determined by thresholding images to include the MitoSOX™ Red fluorescence product or Green fluorescence product for HIF1A or 3,3′-diaminobenzidine (DAB) staining. Mean intensity (optical density; OD) was calculated per image and was divided by tissue area to calculate positivity per area, as previously described [8].

### 2.7. Enzyme-Linked Immunosorbent Assay (ELISA)

Soluble FLT-1 (sFLT1) in culture medium was measured using VEGF receptor 1 (VEGF R1) Quantikine kit (R&D Systems, Minneapolis, MN, USA) following manufacturer’s instructions [8,9].

### 2.8. COX In Situ Enzyme Chemistry

Fresh-frozen cell preparations were washed in three changes of 0.05 M PBS (pH 7.4). The cells were treated for COX enzyme chemistry as described previously [47,48,49,50,51]. Representative digital images of cell culture preparations (Thermo Scientific™: Nunc™ Lab-Tek™ II Chamber Slide™ System) were acquired. Four images were obtained and quantified per sample as replicates. Morphometric measurements were performed as described in Section 2.6. 

### 2.9. Human Subjects 

Details regarding preeclampsia diagnosis and placental collection have been published [9,52]. These human studies were approved by the institutional review board (IRB) at the Beth Israel Deaconess Medical Center, and subjects gave informed consent. All subjects presented to Beth Israel Deaconess Medical Center for delivery. Placental biopsies were obtained within 30 min of delivery and placed in 2% Glutaraldehyde for routine EM tissue processing.

### 2.10. Western Blot Analysis 

Eighty to ninety percent confluent HTR-8/SVneo cells were incubated in 5% serum/RPMI medium without additional growth factors overnight. Cells were stimulated with 100 μM H_2_O_2_, in the presence and absence of 200 μM HMP or MitoTEMPO for 20 h. Cells were washed with cold PBS and lysed with 1× cold cell lysis buffer, composition: mixture of 50 mM Tris Base and Tris-HCl, 150 mM Sodium Chloride, 0.5% Sodium Deoxycholate, 0.1% SDS, 1% Nonidet P-40 Substitute, pH 7.40 ± 0.15 (Boston Bio-Products SKU#: BP-115). The lysis buffer was supplied with protease inhibitors—Roche cOmplete™, Mini, EDTA-free Protease Inhibitor Cocktail (Millipore Sigma 04693159001) and phosphatase inhibitors—Roche PhosSTOP (Millipore Sigma 4906837001). Cell lysates were quantified for protein concentrations, and 15 μg protein was separated in 4–15% SDSPAGE and immunoblotted with antibodies against LC3B (LC3B (D11) XP^®^ Rabbit mAb #3868; Cell Signaling Technology) or an antibody that recognizes beta-actin (beta-Actin antibody (C4): sc-47778, Santa Cruz, Dallas, TX, USA). Densitometry was performed using Image Studio Version 5.2; Acquisition System: Odyssey^®^ CLx Infrared Imaging System by LI-COR.

### 2.11. Statistical Analysis

GraphPad Prism 9.5 statistical software (San Diego, CA, USA) was used. After checking the normality tests (Kolmogorov–Smirnov, Saphiro-Wilk, D’Agostino and Pearson and Anderson–Darling tests), in case of normal distribution, parametric unpaired T-test or analysis of variance (ANOVA) with Tukey’s post hoc test were used. Non-parametric Mann–Whitney-U test or Kruskal–Wallis test with Dunn’s post hoc test were used when distribution was non-normal. Data are presented either as mean ± standard error of mean (SEM) or as median [interquartile range/IQR]. Statistical significance was accepted when *p*-value was less than 0.05 (*p* < 0.05). The used significance symbols are the following: *: *p* < 0.05, **: *p* < 0.01, ***: *p* < 0.001.

## 3. Results

### 3.1. Radical Scavenging Activity of HMP 

**Figure 1:**
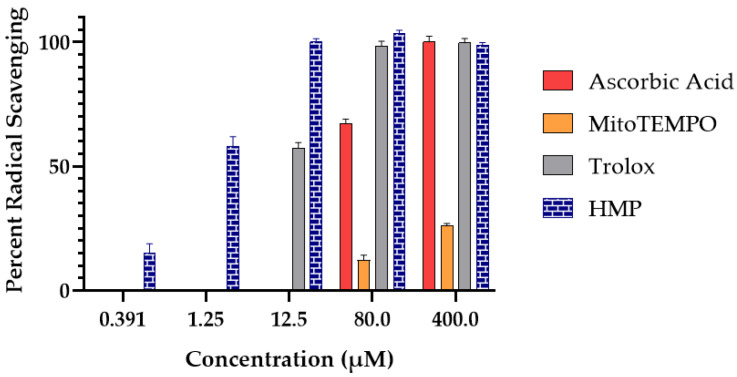
Radical scavenging activity of HMP compared to the antioxidants ascorbic acid, MitoTEMPO and Trolox at different concentrations in the Oxygen Radical Absorbance Capacity (ORAC) assay (*n* = 3 per group).

The Oxygen Radical Antioxidant Capacity (ORAC) assay is a tool for measuring the antioxidant capacity of a compound that scavenges ROS. It measures the fluorescent signal originating from a probe that is quenched in the presence of peroxyl radicals generated in situ by AAPH [53]. The more peroxyl radicals are scavenged by the tested compound, the higher the measured fluorescence intensity will be. The data clearly indicate that HMP shows strong radical scavenging in the ORAC assay, having an EC50/IC50 of 1.32 µM, calculated using the EC50/IC50 equation developed by Sebaugh [54]. HMP is much more potent in this assay than the common antioxidants (ascorbic acid (AA) and Trolox (Vitamin E derivative)) and the mitochondrial-targeted antioxidant MitoTEMPO [42,43,44] (Figure 1). The reference antioxidants showed a significant drop in percent radical scavenging by 12.5 μM, while at that concentration, HMP was still nearly 100% efficacious. HMP at 1.25 μM had an approximately identical percent radical scavenging activity to Trolox at 12.5 μM.

### 3.2. HMP Pre-Treatment Reduced Mitochondrial-Derived ROS Production in H_2_O_2_-Exposed Trophoblast HTR-8/SVneo Cells

To evaluate oxidative stress response in HTR-8/SVneo cells, cells were incubated with increasing concentrations of H_2_O_2_ (40, 60, 80, 100 and 120 μM) for 24 h. The CCK-8 assay was used to assess cell viability, as shown in Supplementary Figure S1. We detected a dose-dependent increase in the cytotoxicity of HTR-8/SVneo cells in response to H_2_O_2_. The IC50 value of H_2_O_2_ concentration in this system was 100 μM (which depicts a 50% killing of the HTR-8/SVneo cells). Based on this finding, in subsequent experiments 100 μM H_2_O_2_ treatment was used to induce oxidative stress. The corresponding sFLT1 levels were also measured and showed a H_2_O_2_ concentration-dependent increase when normalized for viable cell count (Supplementary Figure S2).

**Figure 2:**
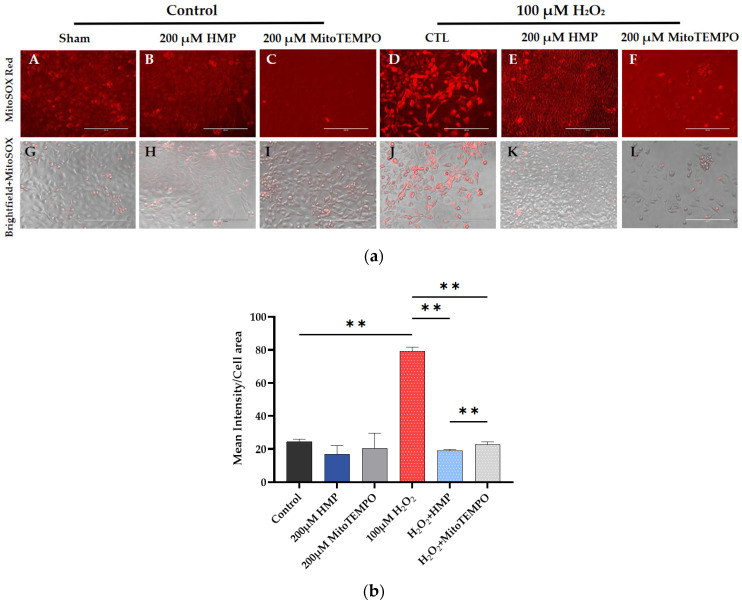
**HMP pre-treatment reduced mitochondrial-derived superoxide production in H_2_O_2_-exposed HTR-8/SVneo trophoblast cells**. (**a**) Representative images from different treatment groups: (**A**–**F**): immunofluorescent and (**G**–**L**): brightfield pictures per group. Bright red color correlates with superoxide production. Bars: 200 μm. (**b**) Quantitation of MitoSOX Red immunofluorescence in trophoblasts: Optical density per area (pixel^2^) of cell surface area was calculated in four high-power fields per sample (*n* = 5 per group). Mann–Whitney-U-test, median [IQR]. Control vs. 100 μM H_2_O_2_: **: *p* < 0.01, 100 μM H_2_O_2_ vs. H_2_O_2_ + HMP: **: *p* < 0.01, 100 μM H_2_O_2_ vs. H_2_O_2_ + MitoTEMPO: **: *p* < 0.01 and H_2_O_2_ + HMP vs. H_2_O_2_ + MitoTEMPO: **: *p* < 0.01.

In subsequent experiments, 100 μM H_2_O_2_ successfully induced mitochondrial-derived oxidative stress as measured by the MitoSox assay: the H_2_O_2_ group had significantly higher intensity (*p* < 0.01) than the control group. This was eliminated by pre-treatment with 200 µM HMP or MitoTEMPO, where there was significantly lower intensity compared to the H_2_O_2_-treated group. HMP caused a significantly greater decrease than MitoTEMPO (*p* < 0.01) (Figure 2a,b).

These data indicate that the initiating factor in the development of preeclampsia, i.e., mitochondrial oxidative stress (superoxide—O^2^—measured by MitoSOX Red), is observed in our H_2_O_2_-induced cell culture model and can be reduced by pre-treatment with HMP.

HMP’s effect in reducing mitochondrial-derived superoxide production is greater than that of the reference antioxidant MitoTEMPO.

### 3.3. HMP Pre-Treatment Reduced HIF1A Expression in H_2_O_2_-Exposed Trophoblast HTR-8/SVneo Cells

We assessed whether the H_2_O_2_-induced oxidative stress upregulated the transcription factor HIF1A in these cells. 

**Figure 3:**
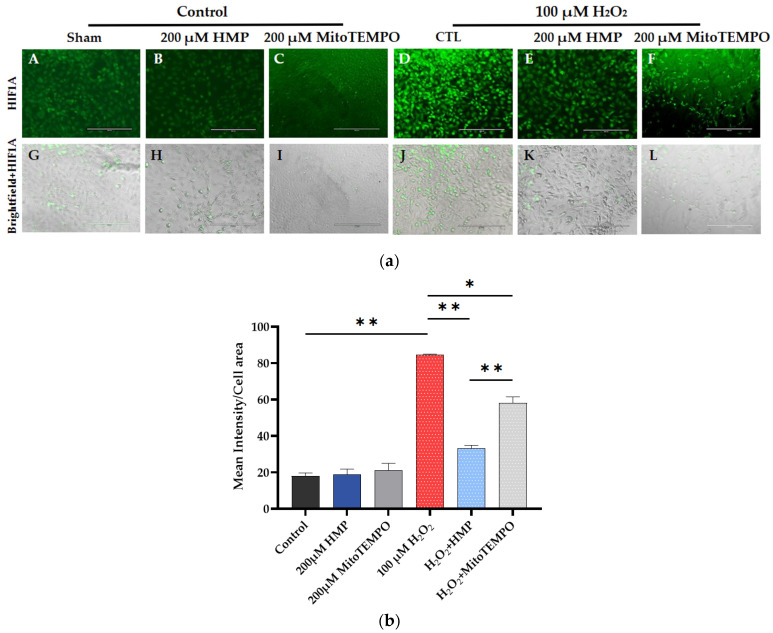
**HMP pre-treatment reduced HF1A expression in H_2_O_2_ -exposed HTR-8/SVneo trophoblast cells**. (**a**) Representative images from different treatment groups: (**A**–**F**): immunofluorescent and (**G**–**L**): brightfield pictures per group. Bright green color correlates with HIF1A expression. Bars: 200 μm. (**b**) Quantitation of HIF1A immunofluorescence in trophoblasts: Optical density per area (pixel^2^) of cell surface area was calculated in four high-power fields per sample (*n* = 5 per group). Mann–Whitney-U test, median [IQR]. Control vs. 100 μM H_2_O_2_: **: *p* < 0.01, 100 μM H_2_O_2_ vs. H_2_O_2_ + HMP: **: *p* < 0.01, 100 μM H_2_O_2_ vs. H_2_O_2_ + MitoTEMPO: *: *p* < 0.05 and H_2_O_2_ + HMP vs. H_2_O_2_ + MitoTEMPO: **: *p* < 0.01.

In H_2_O_2_-induced cells, significantly increased green fluorescent intensity (HIF1A) can be seen compared to control (*p* < 0.01). Pre-treatment of the cells with the antioxidant HMP significantly reduced HIF1A expression (*p* < 0.01 and *p* < 0.05), and did so more efficiently than MitoTEMPO, the reference antioxidant (*p* < 0.01) (Figure 3a,b).

### 3.4. HMP Pre-Treatment Reduced sFLT1 Protein Expression in H_2_O_2_-Exposed Trophoblast HTR-8/SVneo Cells

**Figure 4:**
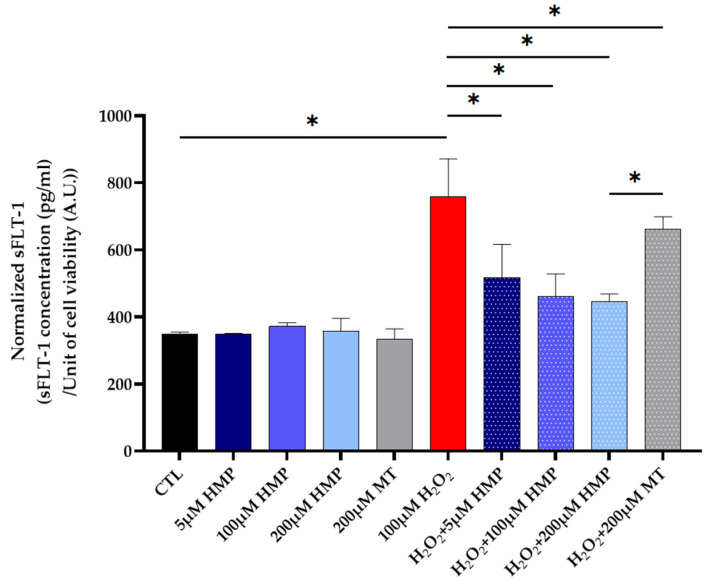
**HMP pre-treatment reduced sFLT1 protein expression in H_2_O_2_-exposed trophoblast HTR-8/SVneo cells.** Normalized sFLT1 data according to different treatment groups (*n* = 4 per group). Abbreviations: CTL = control, MT = MitoTEMPO. Mann–Whitney-U test, median [IQR]. CTL vs. 100 μM H_2_O_2_: *: *p* < 0.05, 100 μM H_2_O_2_ vs. H_2_O_2_ + 5 μM HMP: *: *p* < 0.05, 100 μM H_2_O_2_ vs. H_2_O_2_ + 100 μM HMP: *: *p* < 0.05, 100 μM H_2_O_2_ vs. H_2_O_2_ + 200 μM HMP: *: *p* < 0.05, 100 μM H_2_O_2_ vs. H_2_O_2_ + 200 μM MT: *: *p* < 0.05 and H_2_O_2_ + 200 μM HMP vs. H_2_O_2_ + 200 μM MT: *: *p* < 0.05.

The expression of the anti-angiogenic factor sFLT1 was assessed in the H_2_O_2_-stressed HTR-8/SVneo cells. As expected, the stressed cells showed high sFLT1 production as assessed by ELISA, and HMP pre-treatment dose-dependently reduced sFLT1 protein expression (Figure 4). This is a significant result since sFLT1 is known to induce the cardinal features of preeclampsia in vivo.

In support of these data, we performed experiments in cultures of human villous trophoblast explants. Supplementary Figure S3 shows that hypoxia (2% O_2_) increased sFLT1 production in the villous explant culture and was dose-dependently reduced with HMP pre-treatment. 

### 3.5. HMP Pre-Treatment Improved the Mitochondrial Energetics in H_2_O_2_-Exposed Trophoblast HTR-8/SVneo Cells

So far, we have shown that the antioxidants were successful in reducing sFLT1 via a reduction in HIF1A. However, we were interested in seeing if other pathways were also impacted. Oxidative stress is mainly initiated in the mitochondria within cells; therefore, we assessed the function of the mitochondria in these HTR-8/SVneo cells after H_2_O_2_ treatments. 

**Figure 5:**
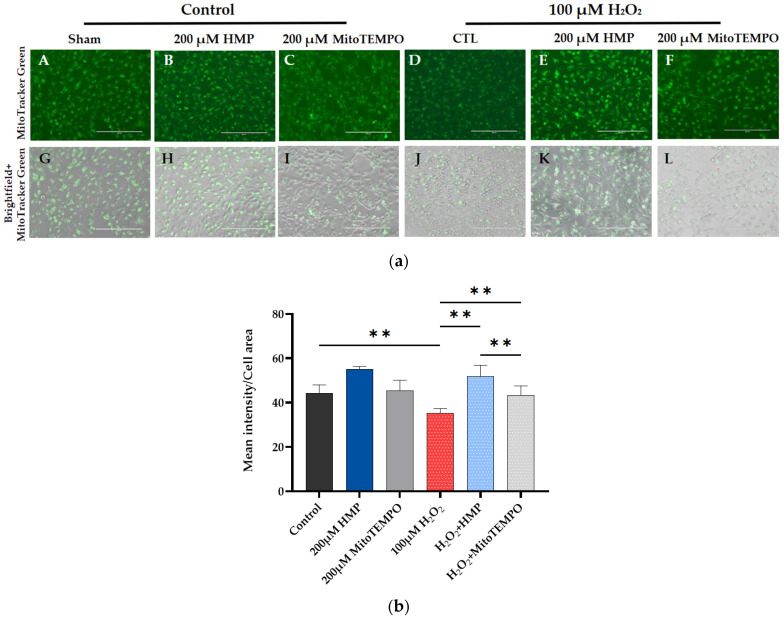
**HMP pre-treatment increased the number of active mitochondria in H_2_O_2_-exposed HTR-8/SVneo trophoblast cells**: (**a**) Representative images from different treatment groups: (**A**–**F**): immunofluorescent and (**G**–**L**): brightfield pictures per group. Bright green color correlates with MitoTracker Green intensity. Bars: 200 μm. (**b**) Quantitation of MitoTracker Green immunofluorescence in trophoblasts: Optical density per area (pixel^2^) of cell surface area was calculated in four high-power fields per sample (*n* = 5 per group). Mann–Whitney-U test, median [IQR]. Control vs. 100 μM H_2_O_2_: **: *p* < 0.01, 100 μM H_2_O_2_ vs. H_2_O_2_ + HMP: **: *p* < 0.01, 100 μM H_2_O_2_ vs. H_2_O_2_ + MitoTEMPO: **: *p* < 0.01 and H_2_O_2_ + HMP vs. H_2_O_2_ + MitoTEMPO: **: *p* < 0.01.

The biomarker for active mitochondria, MitoTracker Green, was significantly reduced in these cells by H_2_O_2_ treatment (*p* < 0.01) (Figure 5a(D,J)). Antioxidant treatment significantly improved mitochondrial activity (Figure 5a(E,K)) (*p* < 0.01), with HMP proving to be more effective than the reference mitochondrial-targeted antioxidant MitoTEMPO (Figure 5a(F,L)) (*p* < 0.01).

### 3.6. HMP Pre-Treatment Improved the Mitochondrial COX Activity in H_2_O_2_-Exposed Trophoblast HTR-8/SVneo Cells

**Figure 6:**
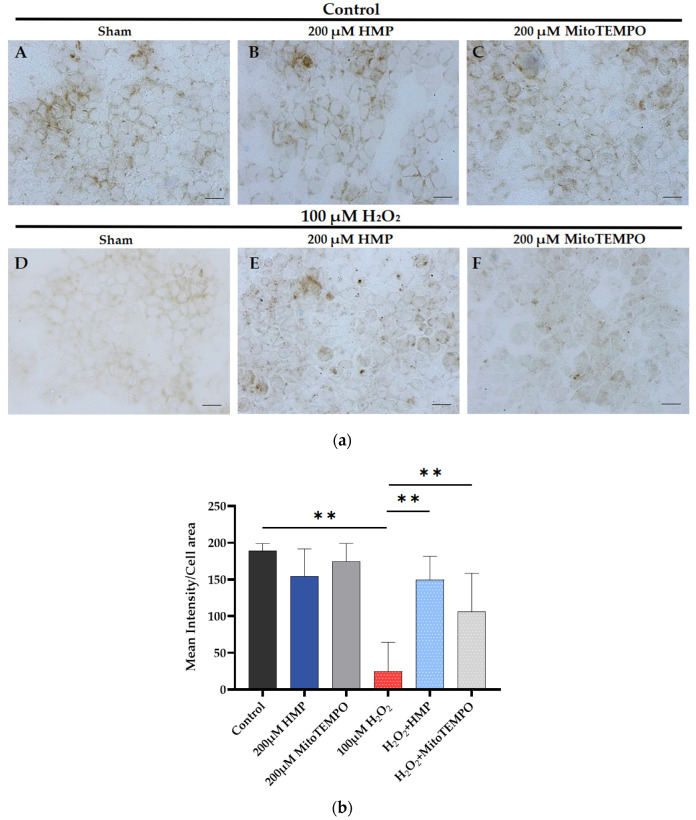
**HMP pre-treatment improved mitochondrial cytochrome C activity in H_2_O_2_-exposed HTR-8/SVneo trophoblast cells**. (**a**) Representative images from different treatment groups: (**A**–**C**): control and antioxidant-treated groups, (**D**–**F**): H_2_O_2_-treated groups. Brown color indicates the COX enzyme activity. Bars: 200 μm. (**b**) Quantitation of COX enzyme activity in trophoblasts: Optical density per area (pixel^2^) of cell surface area was calculated in four high-power fields per sample (*n* = 5 per group). Mann–Whitney-U test, median [IQR]. Control vs. 100 μM H_2_O_2_: **: *p* < 0.01, 100 μM H_2_O_2_ vs. H_2_O_2_ + HMP: **: *p* < 0.01 and 100 μM H_2_O_2_ vs. H_2_O_2_ + MitoTEMPO: **: *p* < 0.01.

The electron transport chain activity can be evaluated by COX enzyme chemistry, and it was significantly decreased in response to H_2_O_2_ treatment (*p* < 0.01) (Figure 6a(D)). COX activity was restored by both HMP and MitoTEMPO pre-treatment, but with better efficiency by HMP (*p* < 0.01) (Figure 6a(E,F)).

### 3.7. Autophagy and Mitochondrial Dysfunction in Human Pregnancy and in HTR-8/SVneo Cells

**Figure 7:**
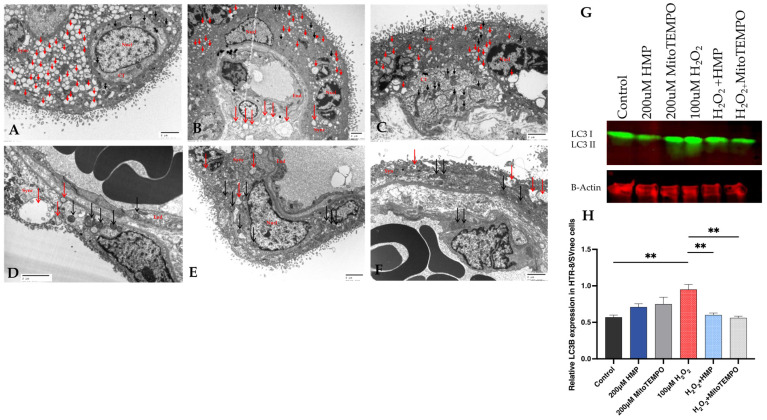
Autophagy and mitochondrial dysfunction in human pregnancy and in HTR-8/Svneo cells. Electron microcopy images of human placental villous tissue from preeclamptic (**A**–**C**) and normal pregnancies (**D**–**F**). Red arrows depict lysosomal autophagy structures which are significantly increased in the preeclamptic syncytiotrophoblast cells compared to control villous tissue. Mitochondria (black arrows) are better preserved in syncytiotrophoblast cells from control pregnancies. (Scale bar: 2 µm) (SCT: syncytiotrophoblast, CT: cytotrophoblast, End: endothelium and Nucl: nucleus). (**G**) Western blot analysis of autophagy markers: LC3B-I and -II (LC3-I and LC3-II in HTR-8/SVneo cells incubated with 100 μM H_2_O_2_ for 20 h in the presence of 200 μM HMP or 200 μM MitoTEMPO. The expression of beta-actin was used as an internal control. (**H**) Densitometry analysis of Western blot shown in (**G**). These experiments were independently performed at least three times (composite result shown in Supplementary Figure S4). Mann–Whitney-U test, median [IQR]. Control vs. 100 μM H_2_O_2_: **: *p* < 0.01, 100 μM H_2_O_2_ vs. H_2_O_2_ + HMP: **: *p* < 0.01 and 100 μM H_2_O_2_ vs. H_2_O_2_ + MitoTEMPO: **: *p* < 0.01.

It is well known that excessive oxidative stress—ROS production—may lead to the covalent modification of proteins and functional changes in trophoblasts, causing an increase in apoptosis and autophagy and finally changes in placental function in pregnancy [55,56,57]. It has been reported that there is increased autophagy in HTR-8/SVneo cells due to oxidative stress and inflammasome activation [58,59]. First, we demonstrated that syncytiotrophoblast autophagy is upregulated in human preeclamptic placenta compared to normal placenta (Figure 7A,F). Next, we examined the autophagy status in H_2_O_2_-exposed trophoblast HTR-8/SVneo cells. H_2_O_2_ upregulated LC3B expression in the cells, which was attenuated by HMP pre-treatment (Figure 7G,H).

## 4. Discussion

In our cell-based assays, pre-treatment with HMP, the nitroxide compound, reduced mitochondrial-derived ROS production in H_2_O_2_-exposed trophoblast cells, indicating that the key factor in the development of PE, oxidative stress, can be alleviated by the antioxidant. HMP also reduced the downstream expression of the transcription factor HIF-1A. Consequently, HMP reduced the expression of anti-angiogenic factor-sFLT1 protein in H_2_O_2_-exposed HTR-8/SVneo cells. HMP also improved the mitochondrial bioenergetics in the stressed HTR-8/SVneo cells, which is another promising characteristic of the applied HMP. 

The presence of oxidative species, such as reactive oxygen and nitrogen species (ROS, RNS), is crucial for cell survival, but an excess can cause cell death. Therefore, the body needs to maintain balanced levels of oxidized and reduced forms of electron carriers in redox homeostasis [60]. An overproduction of ROS and RNS contributes to the development and progression of many ailments, among them PE [61]. Free radical species may not be the defining factor of the disease, but the underlying effect radicals have on the progression of diseases is clear.

The induction of oxidative stress by H_2_O_2_ in our model induced significant mitochondrial superoxide production. Nitroxides are known superoxide dismutase mimetics and, as such, are theoretically regenerated during the catalytic removal of superoxide. Therefore, HMP is a valid compound to use in this model and its superior efficacy over other antioxidants merits its use in in vivo PE models as well.

Hypoxia-inducible factor 1 alpha (HIF1A) is a necessary component of the cellular oxygen-sensing machinery and has been implicated as a major regulator of trophoblast differentiation. Elevated levels of HIF1A in the human placenta have been linked to the development of pregnancy-associated disorders, such as PE. Also, pregnant mice overexpressing HIF-1α have significantly elevated blood pressure and proteinuria. HIF1A transgenic mice show fetal intrauterine growth restriction (IUGR), decreased placental weights and histopathological placental abnormalities; therefore, they can be used as an in vivo model of PE [62]. Our studies showed that exposure of HTR-8/SVneo cells to H_2_O_2_ induces heightened HIF1A expression and this can be prevented by HMP pre-treatment. In line with these results, the subsequent measurement of anti-angiogenic factor sFLT1 expression has corroborated that the effect of HMP on HIF1A also has a downstream effect in reducing sFLT1 expression. The reduction of sFLT1 by HMP has relevance to the in vivo use of this compound as sFLT1 is not only an established biomarker for the development of PE (recently FDA-approved for risk management in PE), but a causative mediator of PE [26,27,28].

ROS production in the placenta in PE pregnancy is well known by our group and others [8,9,10,11,12,13,14,15,16,17,18,19]. In the first trimester, it can be beneficial in inducing signaling pathways that can promote placental angiogenesis, invasion of trophoblasts into spiral arteries and differentiation and transport in normal pregnancy. However, if it is prolonged, then the mitochondria of trophoblast and endothelial cells within the placenta can be damaged as we have shown previously [8,9]. HMP clearly reduced cell injury as assessed by CCK-8 (Figure S1) along with a reduction in MitoSOX Red mitochondrial-derived superoxide production. 

In the pathogenesis of PE, abnormal uteroplacental remodeling leads to placental hypoperfusion, causing fetal growth restriction and pregnancy-related hypertension, which are associated with endothelial dysfunction and reduced vascular nitric oxide (NO) bioavailability. Our studies with HMP have relevance to NO metabolism through its SOD-mimetics effect. Since HMP removes superoxide and therefore prevents its reaction with NO to form peroxynitrite, this will increase NO bioavailability and this is an added benefit of using the nitroxide-type antioxidant. Other examples of NO modulation include tetrahydrobiopterin (BH4), which is a redox cofactor for eNOS (endothelial NO synthase) [63]. Restoration of endothelial cell BH4 with reduced folates identifies a novel therapeutic target for the prevention and treatment of pregnancy-related hypertension such as PE [64]. Two clinical trials referencing this pathway in PE are NCT05847361 and NCT05434195 (Clinical Antenatal Randomised Study to CharactErise Key Roles of TetrahydroFOLate in HyperTensive Pregnancies (CAREFOL-HT)), the latter one involving interventions with patients to act on this specific metabolic cascade. While the restoration of NO bioavailability by HMP may be significant, the major activity of HMP is on mitochondrial function.

The mitochondrial electron transport chain enzyme cytochrome C oxidase (COX) is diminished in the syncytiotrophoblast cells of the placenta, implicating mitochondrial damage/dysfunction as a potential contributor to the pathogenesis of PE [9]. In the present study, mitochondrial COX enzyme activity was reduced in the H_2_O_2_-treated cells, as we have reported in human PE [9], and it is important to note that HMP was beneficial in restoring this function in the HTR-8/SVneo cells.

We examined whether HMP affects autophagy in the stressed HTR-8/SVneo cells and found that the redox modulator normalized autophagy response in these trophoblast cells. This has relevance to human pregnancy since we and others have shown that autophagy can get “out of control” and cause massive cell destruction in the placenta in pathological pregnancies [56]. HMP and other redox modulators may be beneficial in this regard.

## 5. Conclusions

In conclusion, reducing placental trophoblast oxidative stress with nitroxide antioxidants that maintain/restore mitochondrial function presents a potential novel therapeutic approach for the treatment of preeclampsia. These data imply that the use of redox modulators at an early stage has the potential to prevent the trophoblast and endothelial injury that is observed in PE. Future investigation is warranted regarding the in vivo use of this compound.

## Data Availability

The data presented in this study are available on request from the corresponding author.

## Supplementary Materials

The following supporting information can be downloaded at: https://www.mdpi.com/article/10.3390/antiox12081578/s1, Figure S1: Cell viability assay in HTR-8/SVneo cells after exposure to H_2_O_2_ and pre-treatment with HMP; Figure S2: Normalized sFLT-1 expression in HTR-8/SVneo cells exposed to H_2_O_2_ and pretreated with HMP; Figure S3: Normalized sFLT-1 expression in human placental villus explant samples exposed to normoxia and hypoxia and pre-treated with HMP; Figure S4: Western blot analysis of autophagy markers: LC3B-I and -II expression in HTR8/SVneo cells incubated with 100 uM H_2_O_2_ for 20 h in the presence of 200 uM HMP or 200 uM MitoTEMPO. Ref. [52] is cited in Supplementary Materials.
