# Supplementary material for: Nitroxide—HMP—Protects Human Trophoblast HTR-8/SVneo Cells from H2O2-Induced Oxidative Stress by Reducing the HIF1A Signaling Pathway
Source: Antioxidants (Basel). 2023 Aug 8;12(8):1578. doi: 10.3390/antiox12081578 (PMC10451835; doi:10.3390/antiox12081578)
Supplement: Supplementary file 1 [file antioxidants-12-01578-s001.zip › antioxidants-2501048-supplementary.pdf]

antioxidants

Supplemental Methods:

Human Explant Studies, Sample Collection Protocols

Placental samples were collected from patients with normotensive pregnancies that delivered via C-section as described previously [52]. The exclusion criteria for this study of controls were diabetes mellitus, multiple gestation, maternal infection, renal disease, chemical dependency and fetal congenital anomalies. These human studies were approved by the institutional review board (IRB) at the Beth Israel Deaconess Medical Center, and subjects gave informed consent. Briefly, several villous biopsies (2 cm^3) were excised from the maternal surface midway between the chorionic and basal plates, within 30 min of delivery, and the decidual layer was carefully removed. The villous tissue collected was cut in to 0.5 cm^3 and rinsed twice in 50 mL of ice-cold phosphate-buffered saline (PBS) for two minutes. For explant culture, individual clusters of villous trees were dissected under a stereomicroscope and cultured in 1 mL of DMEM/F12 medium containing 10% fetal bovine serum (FBS) and 1% antibiotic-antimycotic (Gibco). Culture medium was supplemented with AKT-1008 or MitoTEMPO to give final concentrations of 10, 50 or 100 μM. The explants were maintained overnight at either 10% or 2% O2 with 5% CO2 at 37 °C. Fresh 0.3 M stock solutions of AKT-1008 were dissolved in DMSO and further diluted in DMEM/F12 media, as recommended by the manufacturer (Akkadian Therapeutics). MitoTEMPO stock solution was produced at 10 mg/mL in sterile water and stored at −20 °C.

Supplemental Figures:

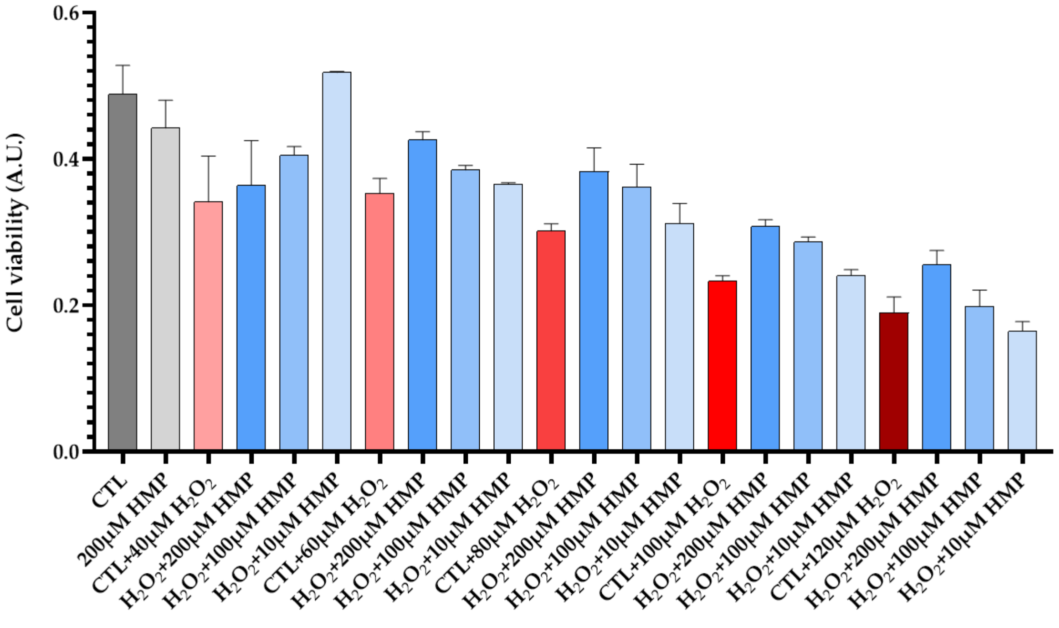

Figure S1. Cell viability assay in HTR-8/SVneo cells after exposure to H_2O_2 and pre-treatment with HMP. Cell viability was tested after varying concentrations of H_2O_2 (40-120 μM) in conjunction with HMP pre-treatment. HTR-8/SVneo cells' viability was tested with Dojindo's CCK8 assay. N = 3 per group. Abbreviations: CTL = control.

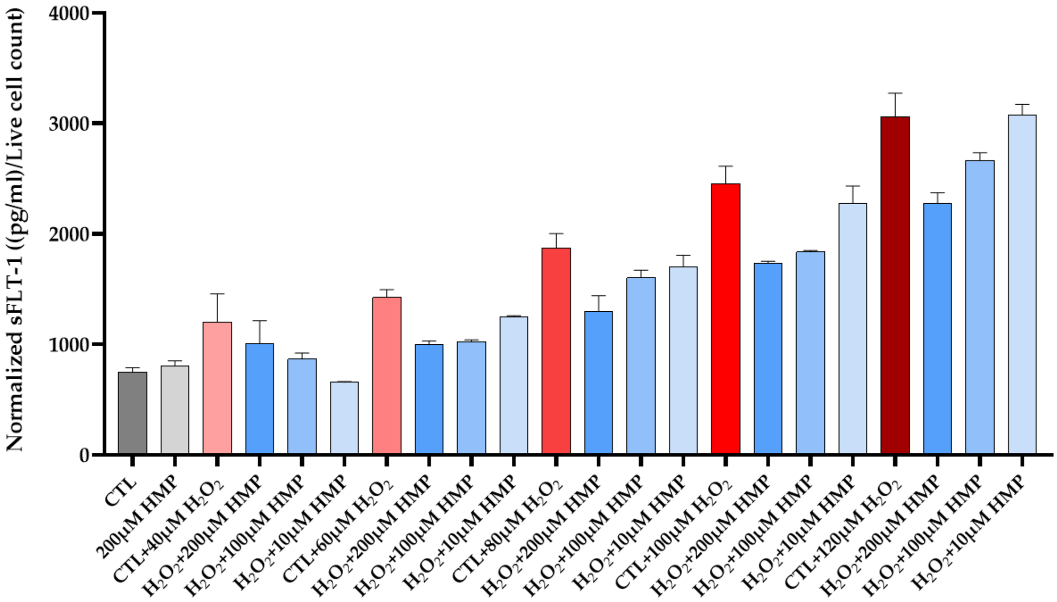

Figure S2. Normalized sFLT-1 expression in HTR-8/SVneo cells exposed to H_2O_2 and pretreated with HMP. sFLT-1 protein expression was analyzed by ELISA and normalized to live cell count in HTR-8 cells exposed to varying concentrations of H_2O_2 (40-120 μM) and pre-treated with HMP (10-200 μM). N = 3 per group. Abbreviations: CTL = control.

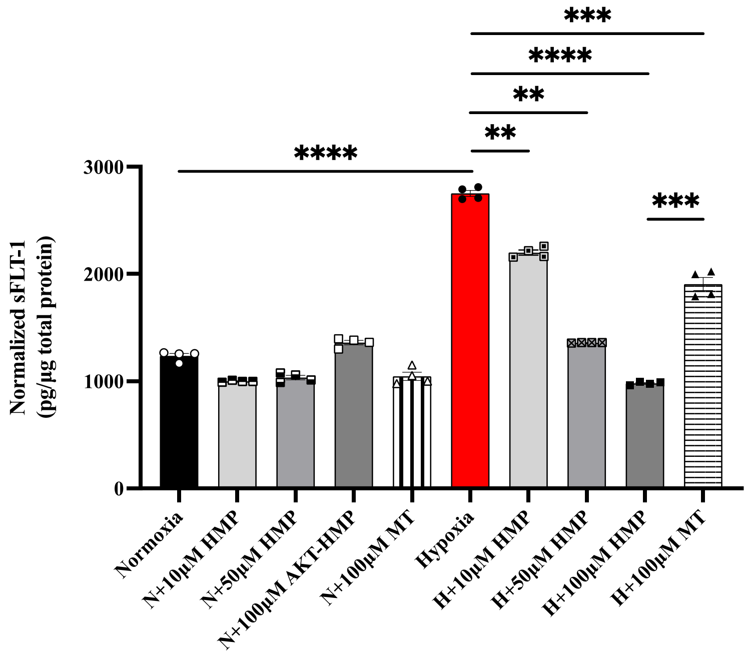

Figure S3. Normalized sFLT-1 expression in human placental villus explant samples exposed to normoxia and hypoxia and pre-treated with HMP. sFLT-1 protein expression was analyzed by ELISA in human villus explant samples exposed to either normoxia or hypoxia and pre-treated with HMP (10-100 uM). N = 4 per group. Abbreviations: N = normoxia, H = hypoxia, MT = MitoTempo. Mann–Whitney-U test, median [IQR]. Normoxia vs. hypoxia: ****: $p < 0.0001$, hypoxia vs. H + 10 uM HMP: **: $p < 0.01$, hypoxia vs. H + 50 uM HMP: **: $p < 0.01$, hypoxia vs. H + 100 uM HMP: ****: $p < 0.0001$, hypoxia vs. 100 uM MT: ***: $p < 0.001$ and H + 100 uM HMP vs. 100 uM MT: ***: $p < 0.001$.

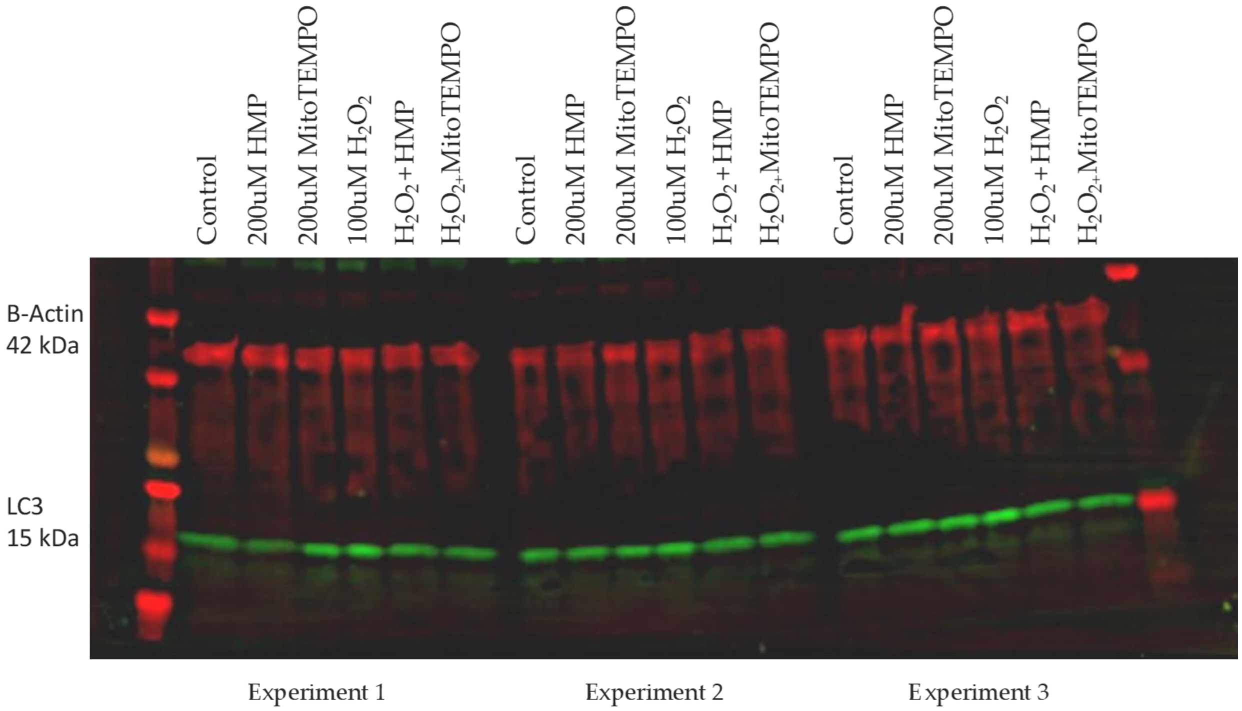

Figure S4. Western blot analysis of autophagy markers: LC3B-I and -II expression in HTR8/SVneo cells incubated with 100uM H_2O_2 for 20 h in the presence of 200 uM HMP or 200 uM MitoTEMPO. The expression of beta-actin was used as an internal control. These experiments were independently performed three times.
